# Vaccine-linked chemotherapy improves cardiac structure and function in a mouse model of chronic Chagas disease

**DOI:** 10.3389/fcimb.2023.1106315

**Published:** 2023-02-09

**Authors:** Kathryn M. Jones, Elise N. Mangin, Corey L. Reynolds, Liliana E. Villanueva, Julio Vladimir Cruz, Leroy Versteeg, Brian Keegan, April Kendricks, Jeroen Pollet, Fabian Gusovsky, Maria Elena Bottazzi, Peter J. Hotez

**Affiliations:** ^1^Texas Children’s Hospital Center for Vaccine Development, Department of Pediatrics, Division of Tropical Medicine, Baylor College of Medicine, Houston, TX, United States; ^2^Department of Molecular Virology and Microbiology, Baylor College of Medicine, Houston, TX, United States; ^3^Department of Molecular Physiology, Baylor College of Medicine, Houston, TX, United States; ^4^Laboratorio de Parasitología, Centro de Investigaciones Regionales Dr. Hideyo Noguchi, Universidad Autónoma de Yucatán, Mérida, Yucatán, Mexico; ^5^Cell Biology and Immunology Group, Wageningen University & Research, Wageningen, Netherlands; ^6^Global Health Research, Eisai, Inc., Cambridge, MA, United States; ^7^Department of Biology, Baylor University, Waco, TX, United States; ^8^James A. Baker III Institute for Public Policy, Rice University, Houston, TX, United States; ^9^Hagler Institute for Advanced Study at Texas A&M University, College Station, TX, United States

**Keywords:** *Trypanosoma cruzi*, Chagas disease, vaccine-linked chemotherapy, cardiomyopathy, cardiac function, inflammation, fibrosis

## Abstract

**Introduction:**

Chagas disease, caused by chronic infection with the protozoan parasite Trypanosoma cruzi, affects 6-7 million people worldwide. The major clinical manifestation of Chagas disease is chronic Chagasic cardiomyopathy (CCC), which encompasses a spectrum of symptoms including arrhythmias, hypertrophy, dilated cardiomyopathy, heart failure, and sudden death. Current treatment is limited to two antiparasitic drugs, benznidazole (BNZ) and nifurtimox, but both have limited efficacy to halt the progression of CCC. We developed a vaccine-linked chemotherapy strategy using our vaccine consisting of recombinant Tc24-C4 protein and a TLR-4 agonist adjuvant in a stable squalene emulsion, in combination with low dose benznidazole treatment. We previously demonstrated in acute infection models that this strategy parasite specific immune responses, and reduced parasite burdens and cardiac pathology. Here, we tested our vaccine-linked chemotherapy strategy in a mouse model of chronic T. cruzi infection to evaluate the effect on cardiac function.

**Methods:**

Female BALB/c mice infected with 500 blood form T. cruzi H1 strain trypomastigotes were treated beginning 70 days after infection with a low dose of BNZ and either low or high dose of vaccine, in both sequential and concurrent treatments streams. Control mice were untreated, or administered only one treatment. Cardiac health was monitored throughout the course of treatment by echocardiography and electrocardiograms. Approximately 8 months after infection, endpoint histopathology was performed to measure cardiac fibrosis and cellular infiltration.

**Results:**

Vaccine-linked chemotherapy improved cardiac function as evidenced by amelioration of altered left ventricular wall thickness, left ventricular diameter, as well as ejection fraction and fractional shortening by approximately 4 months of infection, corresponding to two months after treatment was initiated. At study endpoint, vaccine-linked chemotherapy reduced cardiac cellular infiltration, and induced significantly increased antigen specific IFN-γ and IL-10 release from splenocytes, as well as a trend toward increased IL-17A.

**Discussion:**

These data suggest that vaccine-linked chemotherapy ameliorates changes in cardiac structure and function induced by infection with T. cruzi. Importantly, similar to our acute model, the vaccine-linked chemotherapy strategy induced durable antigen specific immune responses, suggesting the potential for a long lasting protective effect. Future studies will evaluate additional treatments that can further improve cardiac function during chronic infection.

## Introduction

Chagas disease is caused by infection with the protozoal parasite *Trypanosoma cruzi*, affecting approximately 6-7 million people worldwide (Lidani et al., 2019). Infection is mainly spread by contact with feces from an infected hematophagous insect of the Triatomine family, but can also occur through congenital infection, blood transfusion and organ transplant (Rassi et al., 2010). Upon entry into the host, the parasite is capable of invading any nucleated cell where it replicates, then ruptures the cell releasing parasites that circulate in the vasculature and invade cells in any tissues (Machado et al., 2012). Cell damage, as well as pathogen associated molecular patterns from *T. cruzi*, incite the host inflammatory immune response to control parasite levels (Oliveira et al., 2004). However, the host inflammatory immune response is only able to significantly reduce parasite levels, not eliminate all parasites (Añez et al., 1999; Zhang and Tarleton, 1999). Thereafter, persistence of parasites in tissues leads to chronic inflammation, progressive fibrosis, edema, and hypertrophy (Bonney and Engman, 2008; Bonney et al., 2019). Clinically, infected individuals may develop a self-limiting illness with nonspecific flu-like symptoms during the first 1-2 months after infection, but resolution of symptoms without treatment coincides with significant reduction of parasite levels by the inflammatory immune response (Machado et al., 2012). Subsequently, individuals enter the chronic infection phase where they may remain clinically asymptomatic for years to decades (Machado et al., 2012). Approximately 60-70% of chronically infected individuals remain asymptomatic for life and are considered indeterminate (Machado et al., 2012). The remaining 30-40% develop clinical disease, with approximately 75% of those individuals developing cardiac symptoms and the remaining 25% developing gastrointestinal disease (Rassi et al., 2010). Chronic Chagasic cardiomyopathy (CCC) is the most severe manifestation of chronic *T. cruzi* infection, resulting in 2.3 million disability adjusted life years (DALYs) and can incur an annual cost of $11,000 per patient for treatment ([[NoAuthor]]; Lee et al., 2012). Thus, there is an urgent need for treatment strategies that can alleviate disease burdens.

Clinical manifestations of CCC encompass a spectrum of symptoms, including mild to severe arrhythmias, apical aneurysms, ventricular dilation, heart failure and sudden death (Ribeiro et al., 2012). Symptoms arise due to the *T. cruzi* induced inflammatory and fibrotic tissue damage which leads to cardiac remodeling (Cruz et al., 2017). Histologic studies of cardiac tissue from CCC patients have shown that CD8+ and CD4+ T lymphocytes are the predominant infiltrating leukocyte population in cardiac tissue, and increased levels of pro-inflammatory cytokines IFN-γ, TNF-α, and IL-6 are also present (Higuchi Mde et al., 1993; Higuchi et al., 1997; Reis et al., 1997). Additionally, progression of cardiac fibrosis correlates with progression of disease severity (Noya-Rabelo et al., 2018; Senra et al., 2018). Thus, the central problem to addressing cardiac disease due to chronic *T. cruzi* infection is the fact that specific treatment is limited to only two anti-parasitic drugs, benznidazole (BNZ) and nifurtimox, which act to directly kill parasites peripherally but may not significantly reduce the parasite burden in the heart, nor ameliorate host tissue damage (Viotti et al., 2006; Jackson et al., 2010; Urbina, 2010). CCC can ensue due to the progression of host inflammation and fibrosis. Thus, while both drugs have high efficacy during acute infection (Viotti et al., 2006), a large, multi-center clinical trial demonstrated that in patients with established cardiac disease, BNZ failed to prevent the progression of CCC and ultimately, cardiac death (Morillo et al., 2015). Further complicating the limited efficacy of BNZ is the fact that intolerable side effects occur in 40% of patients, resulting in early termination of treatment (Viotti et al., 2009; Molina et al., 2015). Thus, alternative therapies with improved efficacy, reduced adverse effects, and targeting the deleterious host responses are urgently needed.

Vaccines are an attractive option to address the need for improved therapies for Chagas disease. Studies comparing parasite specific immune responses between indeterminate and CCC patients have revealed that a balanced T_H_1/T_H_2/T_H_17 cytokine profile, with increased levels of IFN-γ, IL-10 and IL-17A, correlate with preserved cardiac function, while patients with CCC have predominantly a Th1 response with higher levels of IFN-γ, IL-6, and TNF-α and little IL-10 and IL-17A (Laucella et al., 2004; Magalhaes et al., 2013; Sousa et al., 2014). Building on this knowledge, researchers have developed numerous vaccine candidates that target specific parasite antigens and modulate the host immune responses (Rios et al., 2019; Bivona et al., 2020). One promising vaccine candidate is the *T. cruzi* flagellar calcium binding protein designated Tc24 (Dumonteil et al., 2012). This antigen is located along the inner leaflet of the flagellar membrane and is highly expressed in the extracellular trypomastigote form as well as in the non-dividing intracellular amastigote form of *T. cruzi* (Versteeg et al., 2021). Several studies have demonstrated that recombinant protein based vaccines containing Tc24 antigens induce strong immune responses, with increased production of antigen specific CD8+ T cells as well as production of IFN-γ, IL-10 and IL-17A (Barry et al., 2016; Seid et al., 2016; Jones et al., 2018; Cruz-Chan et al., 2021). Importantly, Tc24 based vaccines can reduce parasite burdens and tissue damage when used therapeutically in both acute and chronic mouse models of infection (Barry et al., 2019; Cruz-Chan et al., 2021). Additionally, we have constructed a mutated form of Tc24, designated Tc24-C4, with improved stability suitable for large scale expression and purification processes necessary to develop vaccines for clinical use (Seid et al., 2017; Biter et al., 2018). Therefore, Tc24-C4 based vaccines are a promising candidate to address clinical disease.

Vaccine-linked chemotherapy has been proposed as an improved treatment strategy for parasitic diseases to both reduce infection and reduce morbidity (Bergquist et al., 2005; von Seidlein et al., 2020). We were the first to demonstrate in mouse models of acute infection that vaccine-linked chemotherapy using a low dose of benznidazole and a low dose of vaccine, consisting of a stable squalene emulsion containing recombinant Tc24-C4 protein and a TLR4 agonist adjuvant, either E6020 or glucopyranosyl lipid A (GLA), which leads to greater reductions in parasite burdens, cardiac inflammation, and cardiac fibrosis when compared to BNZ or vaccine only treatment (Jones et al., 2018; Cruz-Chan et al., 2021). Further, the vaccine induced a balanced T_H_1/T_H_2/T_H_17 immune response (Cruz-Chan et al., 2021). More recently, vaccine-linked chemotherapy strategies using recombinant trans-sialidase protein (TSA-1) alone, or TSA1-C4 combined with Tc24-C4 protein, have been tested in mouse models of chronic infection and shown to also reduce tissue pathology (Dzul-Huchim et al., 2022; Prochetto et al., 2022). What we had not yet tested and proved was whether our vaccine-linked chemotherapy strategy improved cardiac function during chronic *T. cruzi* infection. We previously showed in acute infection models that both sequential and concurrent treatment with a low dose of BNZ combined with the Tc24-C4/E6020 SE significantly reduced cardiac pathology and parasite burdens. Therefore, in the study reported here we tested both sequential and concurrent vaccine-linked chemotherapy strategies and we evaluated left ventricular wall thickness, chamber size and volume over time in an effort to better characterize the changes in cardiac structure induced by chronic *T. cruzi* infection

## Materials and methods

### Ethics statement

Animal experiments were performed in full compliance with the Public Health Service Policy and the National Institutes of Health Guide for the Care and Use of Laboratory Animals, 8th edition, under a protocol approved by Baylor College of Medicine’s Institutional Animal Care and Use Committee, assurance number and D16-00475 (Council, 2011).

### Mice and parasites

Female BALB/c mice (BALB/cAnNTac) were obtained at 5-6 weeks of age from Taconic (Taconic Biosciences, Inc) and allowed to acclimate for one week prior to studies. Mice were housed in groups of 5 in small microisolator caging, with ad libitum food and water and a 12hr light/dark cycle. *T. cruzi* H1 parasites, originally isolated from a human case in Yucatan, Mexico were maintained by serial passage in female BALB/c mice every 25 to 28 days (Dumonteil et al., 2003; Ruiz-Sanchez et al., 2005).

### Benznidazole

Benznidazole (BNZ) powder (Laborotorio ELEA) was resuspended in 5% DMSO/95% HPMC (0.5% hydroxypropylmethylcellulose/0.4% Tween 80/0.5% benzyl alcohol in deionized water) to a final concentration of 10mg/mL. Benznidazole was administered by oral gavage once daily for 20 days on the schedule described below.

### Vaccine formulations

Recombinant Tc24-C4 protein was expressed and purified according to previously published protocols (Seid et al., 2017). The TLR4 agonist adjuvant E6020 (Eisai, Inc) was dissolved in a stable squalene emulsion (SE). Vaccine formulations comprised of the selected dose of recombinant Tc24-C4 protein and E6020 in 100 µL of a 2% squalene emulsion in 1x PBS pH 7.4 were freshly prepared and mixed just before administration.

### Echocardiography and electrocardiograms

Mice were anesthetized by inhalation of 2-3% isoflurane delivered by precision vaporizer. Fur from the ventral thorax of anesthetized mice was removed with depilatory cream, then mice were positioned in dorsal recumbency on a temperature regulated stage set at 37°C. Core body temperature and heart rate were monitored by rectal thermometer and Doppler electrocardiogram. Prewarmed ultrasound gel was applied to the thorax, and short axis images of the left ventricle were obtained from the left parasternal window with a Vevo 2100 imaging system (FujiFilms Visualsonics, Inc.). M-mode images were obtained at the papillary level to determine left ventricular chamber dimensions and wall thickness. Immediately after echocardiographic evaluation, mice were transferred to a Rodent Surgical Monitor (Mouse Monitor, Indus Instruments) to obtain Lead II electrocardiograms. M-mode images were analyzed using VevoLab software (Fujifilm Visualsonics) to measure left ventricular wall thickness and left ventricle chamber dimensions. Electrocardiogram tracings were analyzed using LabChart Pro software to measure conduction intervals and wave amplitudes.

### Study design

**Figure 1:**
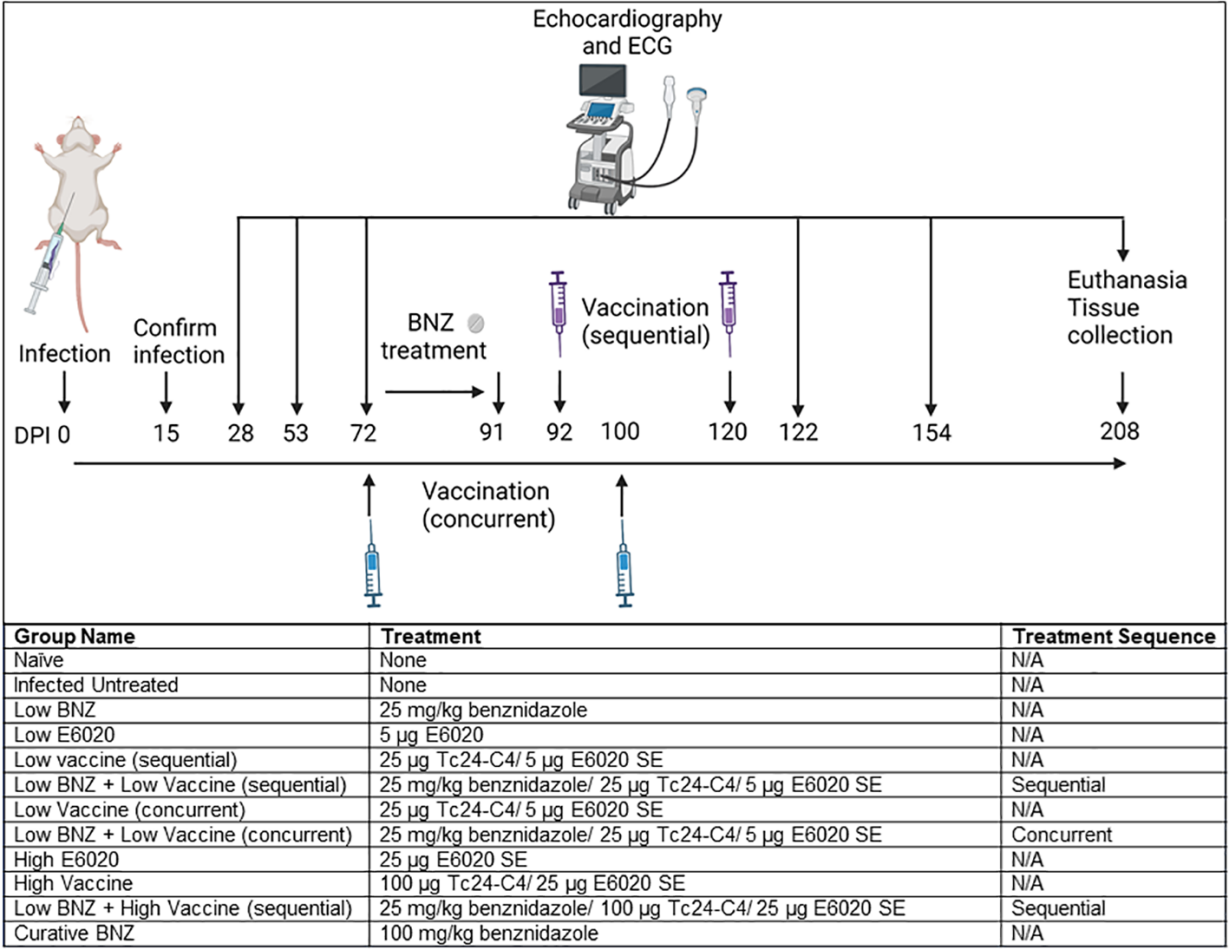
Study Timeline and experimental treatment groups. Mice were infected by intraperitoneal injection at day 0. Infection was confirmed at 15dpi, then echocardiography and electrocardiograms (ECG) were performed at 28dpi, 53dpi and 70dpi. Mice randomly assigned to treatment groups as indicated were treated beginning 70dpi and follow up echocardiography and ECGs were performed at approximately 122dpi, 154dpi, and 208dpi. All mice were euthanized at approximately 209dpi. Image created with Biorender.

All mice were infected on day 0 with 500 blood form *T. cruzi* H1 trypomastigotes by intraperitoneal injection. Naïve age matched mice were left uninfected as controls. Blood was collected by tail vein microsampling from all mice at approximately 15 days post infection (DPI) to confirm parasitemia by quantitative PCR. Echocardiography and electrocardiograms were performed on a subset of naïve and infected mice (n=10 per group) at 28 DPI and 53 DPI. Echocardiography and electrocardiograms were performed on all mice at approximately 72 DPI, then mice were randomly assigned to treatment groups of 8-14 mice per group as described in **Figure 1**. Benznidazole treatments were administered once daily by oral gavage beginning approximately 72 DPI. Vaccinations were administered by subcutaneous injection at approximately 72 DPI and 100 DPI for mice receiving concurrent treatment, and approximately 92 DPI and120 DPI for mice receiving sequential treatment (**Figure 1**). Post treatment echocardiography and electrocardiograms were performed at approximately 122 DPI, 154 DPI, and 208 DPI. Mice were monitored daily for morbidity and any mice that reached humane endpoints were humanely euthanized by anesthetic overdose followed by exsanguination. At the study endpoint, approximately 209 DPI, all mice were humanely euthanized and hearts, whole blood, and spleens were collected for analysis.

### Evaluation of parasite burdens

Parasite burdens were measured in blood and tissue as described (Jones et al., 2018). Briefly, total genomic DNA was isolated from whole blood and cardiac tissue using DNEasy 96 blood and tissue kits (Qiagen) following the manufacturer protocol. Quantitative real time PCR was performed using 4ng of DNA from blood or 50ng of DNA from cardiac tissue using TaqMan Fast Advanced Master Mix (Life Technologies) and oligonucleotides specific for *T. cruzi* nuclear satellite DNA (primers 5’ ASTCGGCTGATCGTTTTCGA 3’ and 5’ AATTCCTCCAAGCAGCGGATA 3’, probe 5’ 6-FAM CACACACTGGACACCAA MGB 3’, Life Technologies). Parasite equivalents were calculated from a standard curve.

### Splenocyte restimulation for immune evaluation

To prepare single cell splenocyte suspensions, spleens were mechanically dissociated through 70µm cell strainers, red blood cells were lysed with ACK lysis solution (Gibco), then washed with RPMI supplemented with 10%FBS/1X Pen/Strep and L-Glutamine (cRPMI). Live splenocytes were quantified using a Cellometer Auto 2000 and AOPI live/dead dye (Nexcelom), then adjusted to a final concentration of 1x10^7^ cells/mL in cRPMI. For each sample, 1x10^6^ live splenocytes were restimulated for 96 hours with 100 µg/mL recombinant Tc24-C4 protein or cRPMI (unstimulated) at 37°C, 5% CO_2._ As a positive control, splenocytes incubated for 6 hours with 20ng/mL phorbol myristate acetate (PMA) (Sigma-Aldrich)/1mg/mL Ionomycin (Sigma-Aldrich) were included.

### Evaluation of cytokine producing splenocytes

To measure secreted cytokines, restimulated splenocytes were harvested and centrifuged at 300 x g for 5 minutes at 4°C to pellet cells, and supernatants were harvested and frozen at -80°C for later analysis. Secreted levels of IL-4, IL-5, IL-6, IL-10, IL-17A and IFN-γ were measured from both unstimulated and Tc24-C4 protein restimulated cells by Luminex using a multiplexed cytokine kit (BioRad) and wall less 96 well plate (Curiox) (Versteeg et al., 2017). To evaluate antigen specific responses, for each sample the value measured from the unstimulated cells was subtracted from the value measured from Tc24-C4 protein stimulated cells.

To quantify antigen specific cytokine producing CD4+ and CD8+ T cells, splenocytes were restimulated as described with the addition of 4.1µg/mL Brefeldin A (BD Biosciences) for the last 6 hours of incubation. Restimulated splenocytes were collected, washed with 1 X PBS, and stained with Live/Dead fixable blue viability dye, anti-CD3e FITC clone 145-2C11 (eBioscience), anti-CD4 Alexa Fluor^®^ 700 clone RM4-5 (eBioscience) and anti CD8a PerCP-Cy5.5 clone 53-6.7 (BD Bioscience). Splenocytes were then fixed with Cytofix/Cytoperm (BD Biosciences) and permeabilized following manufacturer instructions. Permeabilized splenocytes were stained with anti-IFN-γ APC clone XMG1.2 (eBioscience) and anti-IL-4 PE-Cy7 clone BVD6-24G2 (eBioscience). Samples were acquired on a LSR Fortessa Cell Analyzer (BD Biosciences) and 100,000 total events in a live gate were analyzed using FlowJo 8.7 software. To evaluate antigen specific responses the ratio of antigen specific cells to unstimulated cells was calculated by dividing the percent of antigen stimulated cells by the percent of media stimulated cells for each sample.

### Histopathology

Sections of left ventricular heart tissue were fixed in 10% neutral buffered formalin and routinely processed for paraffin embedding and sectioning. Sections were stained with either hematoxylin and eosin (H&E), or Masson’s Trichrome stain. Images of three to five representative sections of the left ventricle from each mouse were captured at 100X magnification using an Amscope microscope for H&E Images, and a Micromaster microscope (Fisher Scientific) for Masson’s Trichrome images. Images were analyzed using ImageJ software (National Institutes of Health) to quantify the number of nuclei per millimeter^2^ of tissue from H&E stained sections, and the percentage of collagen per millimeter^2^ of tissue from Masson’s Trichrome stained sections.

### Statistical analysis

For each parameter measured, data was plotted using GraphPad Prism software (GraphPad). Groups were analyzed for normality, then parametric or non-parametric statistical analyses were performed as appropriate. For each cardiac monitoring parameters on echocardiography measured at multiple timepoints, repeated measures or mixed effects analysis was performed for individual mice within a group to determine if individual mice had significant changes over time after treatment was initiated. For all other parameters measured at a single timepoint, treatments were compared to infected untreated controls using a Kruskal-Wallis one way ANOVA and Dunn’s multiple comparisons test. When comparing the two groups, a Mann-Whitney test was used. P values ≤ 0.05 were considered significant.

## Results

### Vaccine-linked chemotherapy improves *T. cruzi* induced changes in cardiac structure and function

**Figure 2:**
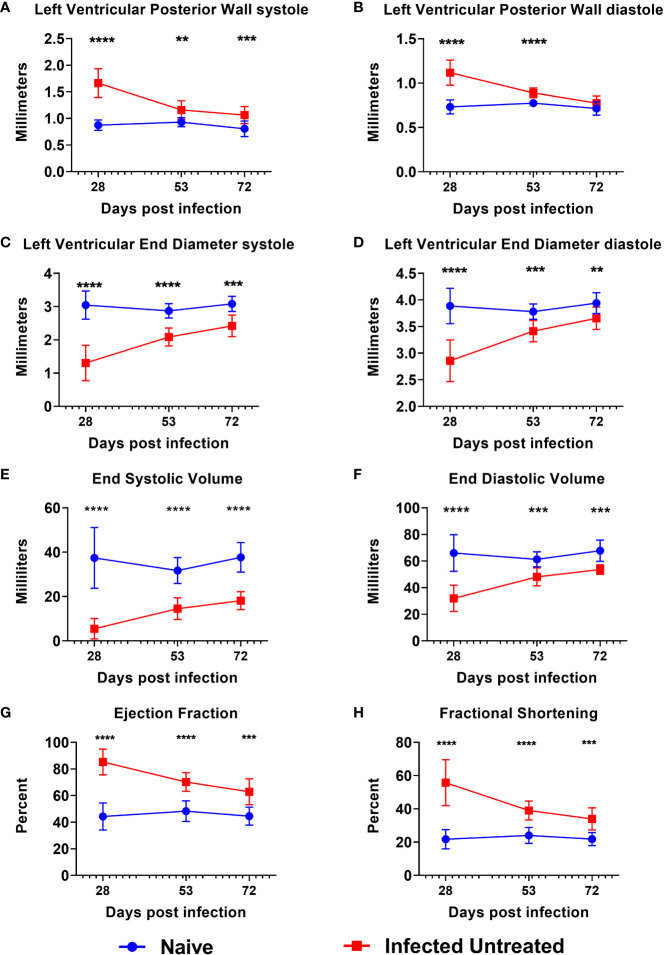
Echocardiography evaluation prior to treatment initiation. M-mode images were used to measure parameters at the end of both systole and diastole, including left ventricular posterior wall thickness **(A, B)**, left ventricular end diameter **(C, D)**, left ventricular volume **(E, F)**, ejection fraction **(G)** and fractional shortening **(H)** at approximately 28dpi, 53dpi and 70dpi. At each timepoint, infected untreated mice were compared to naïve age matched controls*, p≤0.05; **, p≤0.01; ***, p≤0.001; ****, p≤0.0001.

By 15 DPI, almost all mice were confirmed parasitemic (**Supplemental Figure 1**), and any mice that were negative by qPCR were removed from the study. Acute *T. cruzi* infection caused a significant increase in the left ventricular posterior wall thickness (LVPW) compared to age matched naïve mice by 28 DPI during both systole (**Figure 2A**) and diastole (**Figure 2B**). LVPW in infected mice decreased by 53 DPI and 72 DPI in infected mice, but still remained significantly higher than naïve age matched controls (**Figures 2A, B**). Concurrently, acute infection caused a significant decrease in Left Ventricular End Diameter during both systole (LVEDs) (**Figure 2C**) and diastole (LVEDd) (**Figure 2D**), as well as a significant decrease in left ventricular End Systolic Volume (ESV) (**Figure 2E**) and End Diastolic Volume (EDV) (**Figure 2F**) by 28 DPI. Left ventricular diameter and volume in infected mice increased by 53 DPI and 72 DPI, but still remained significantly lower than naïve age matched controls at both time points (**Figures 2C–F**). Finally, *T. cruzi* infection induced significant increases in left ventricular ejection fraction (**Figure 2G**) and fractional shortening (**Figure 2H**) by 28 DPI, and while these parameters decreased by 53 DPI and 72 DPI, they did not reach the levels of naïve age matched controls by 72 DPI (**Figures 2G, H**). Together, these data indicate that acute *T. cruzi* infection induces significant changes in cardiac structure and function and these changes are not completely resolved by the early chronic stage of infection.

**Figure 3:**
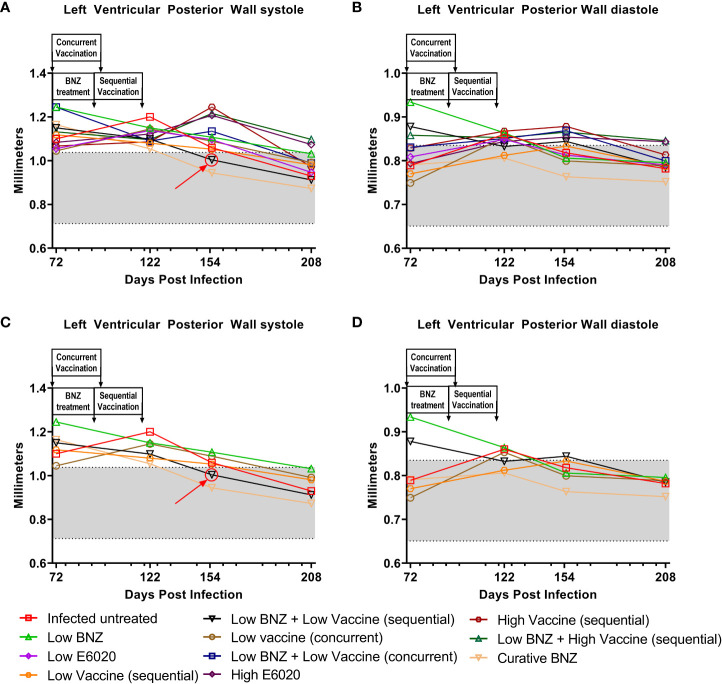
Left ventricular posterior wall thickness (LVPW) after treatment. M-mode images from echocardiographic examination were used to measure LVPW in infected untreated and treated mice at 70dpi, 122dpi, 154dpi and 208dpi during both systole **(A)** and diastole **(B)**. Panels **(C)** and **(D)** illustrate only a subset of treatment groups to better visualize treatment differences between key groups. The grey shaded area represents the mean ± SD of the LVPWs and LVPWd measurement from naïve age matched controls.

**Figure 4:**
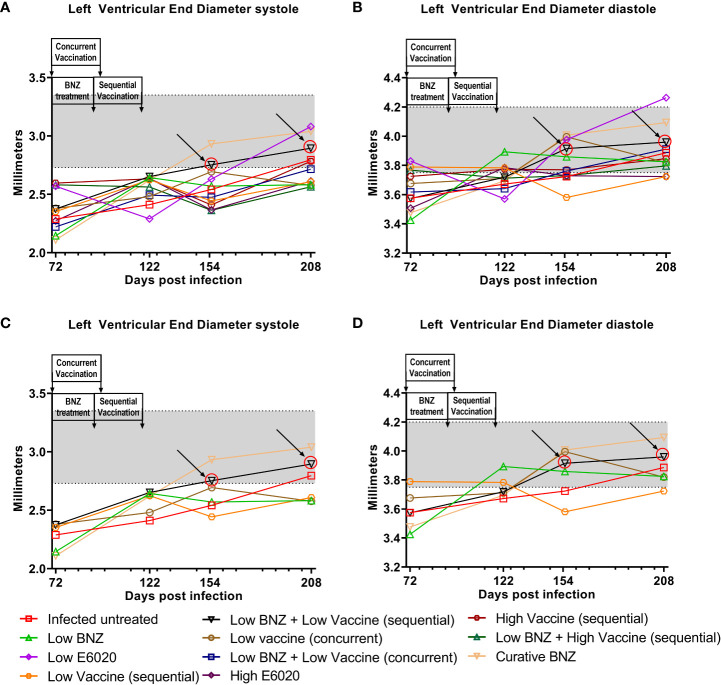
Left Ventricular End diameter (LVED) after treatment. M-mode images from echocardiographic examination were used to measure LVED in naïve, infected untreated and treated mice at 70dpi, 122dpi, 154dpi and 208dpi during both systole **(A)** and diastole **(B)**. Panels **(C)** and **(D)** illustrate only a subset of treatment groups to better visualize treatment differences between key groups. The grey shaded area represents the mean ± SD of the LVEDs and LVEDd measurement from naïve age matched controls.

**Figure 5:**
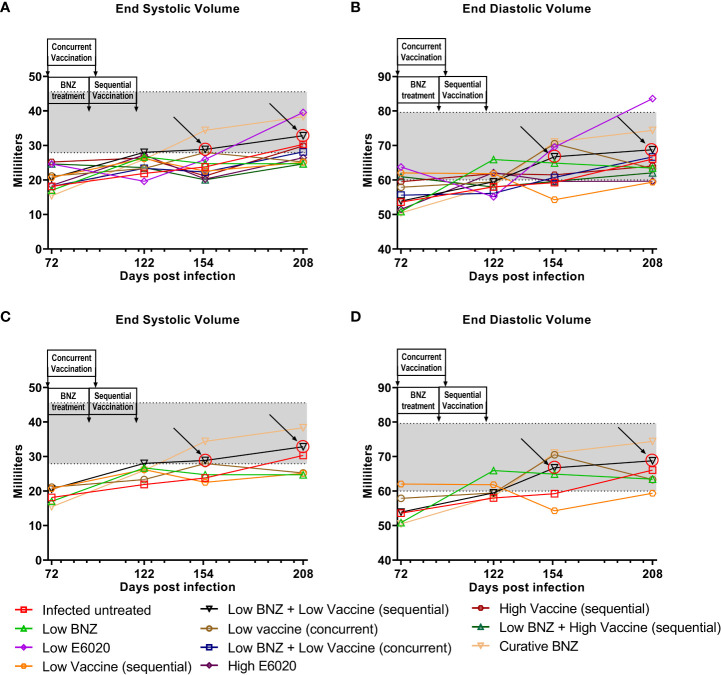
Left ventricular volume after treatment. M-mode images from echocardiographic examination were used to measure left ventricular volume (ESV and EDV) in naïve, infected untreated and treated mice at 70dpi, 122dpi, 154dpi and 208dpi during both systole **(A)** and diastole **(B)**. Panels **(C)** and **(D)** illustrate only a subset of treatment groups to better visualize treatment differences between key groups. The grey shaded area represents the mean ± SD of the ESV and EDV measurement from naïve age matched controls.

To evaluate whether individual treatments significantly changed specific measurements of left ventricular structure, measurements taken from both infected untreated and treated mice at 122 DPI, 154 DPI and 208 DPI were compared to measurements taken at 70 DPI, before treatment was initiated for each individual animal. Infected untreated mice did not have significant changes in LVPW over the course of the study during systole (**Figure 3A**) or diastole (**Figure 3B**). Sequential treatment with Low BNZ + Low vaccine significantly reduced LVPW thickness by 154 DPI during systole (**Figures 3A, C**) when compared to prior to treatment at 70 DPI (**Table 1**). As expected, curative BNZ treatment significantly reduced LVPW by 154 DPI during both systole (**Figures 3A, C**) and diastole (**Figures 3B, D**) and this persisted through 208 DPI (**Table 1**). Interestingly, the low dose vaccine administered at 70 DPI resulted in a significant increase in LVPW during diastole at 122 DPI (**Table 1**). Concurrent with the significantly increased LVPW, infection caused significant decreases in left ventricular end diameter during systole (LVEDs) (**Figures 4A, C**) and diastole (LVEDd) (**Figures 4B, D**), as well as significant decreases in left ventricular volume at the end of systole (ESV) (**Figures 5A, C**) and diastole (EDV) (**Figures 5B, D**) prior to treatment. Sequential treatment with Low BNZ + Low vaccine significantly increased LVEDs (**Figures 5A, C**) and LVEDd (**Figures 5B, D**) by 154 DPI and this change persisted through and 208 DPI (**Table 2**). Curative BNZ treatment also significantly increased LVEDs (**Figures 5A, C**) and LVEDd (**Figures 5B, D**) as early as 122 DPI, and this persisted through 154 DPI and 208 DPI (**Table 2**, **Figure 5**). Similarly, sequential treatment with Low BNZ + Low Vaccine significantly increased left ventricular volume during systole (ESV) by at 208 DPI (**Figures 5A, C**) and diastole (EDV) by 154 DPI and persisting through 208 DPI (**Figures 5B, D**, **Table 3**). Similarly, concurrent treatment with Low BNZ + Low Vaccine significantly reduced ESV at 208 DPI (**Table 3**). Again, the curative dose of BNZ significantly increased ESV and EDV at 122 DPI, 154 DPI and 208 DPI (**Figure 5**, **Table 3**). Together, these data indicate that sequential vaccine-linked chemotherapy improve infection induced changes in cardiac structure.

**Table 1:** Left ventricular posterior wall thickness.

	LVPWs				LVPWd			
Days post infection	72	122	154	208	72	122	154	208
**Naive**	0.806 ± 0.146	0.912 ± 0.157	0.851 ± 0.196	0.932 ± 0.135	0.715 ± 0.075	0.742 ± 0.069	0.738 ± 0.097	0.777 ± 0.122
**Infected untreated**	1.100 ± 0.149	1.200 ± 0.154	1.059 ± 0.162	0.930 ± 0.168	0.789 ± 0.084	0.8602 ± 0.071	0.818 ± 0.096	0.782 ± 0.110
**Low BNZ**	1.245 ± 0.288	1.150 ± 0.312	1.107 ± 0.146	1.032 ± 0.143	0.934 ± 0.340	0.864 ± 0.185	0.806 ± 0.095	0.796 ± 0.074
**Low E6020**	1.055 ± 0.117	1.138 ± 0.132	1.096 ± 0.118	0.947 ± 0.217	0.809 ± 0.083	0.849 ± 0.065	0.813 ± 0.076	0.790 ± 0.189
**Low Vaccine (sequential)**	1.118 ± 0.141	1.080 ± 0.163	1.050 ± 0.126	0.981 ± 0.164	0.770 ± 0.070	0.812 ± 0.045	0.833 ± 0.088	0.787 ± 0.091
**Low BNZ + Low Vaccine (sequential)**	1.150 ± 0.152	1.098 ± 0.221	1.004 ± 0.114	0.912 ± 0.133**	0.878 ± 0.0941	0.8326 ± 0.050	0.844 ± 0.113	0.785 ± 0.045*
**Low vaccine (concurrent)**	1.045 ± 0.123	1.145 ± 0.163	1.089 ± 0.205	0.991 ± 0.202	0.749 ± 0.056	0.853 ± 0.058****	0.800 ± 0.089	0.788 ± 0.083**$**
**Low BNZ + Low Vaccine (concurrent)**	1.244 ± 0.226	1.090 ± 0.171	1.135 ± 0.180	0.988 ± 0.196*	0.831 ± 0.086	0.851 ± 0.080	0.869 ± 0.102	0.799 ± 0.104
**High E6020**	1.083 ± 0.225	1.122 ± 0.244	1.207 ± 0.201	1.074 ± 0.174	0.794 ± 0.124	0.844 ± 0.075	0.854 ± 0.053	0.843 ± 0.099
**High Vaccine (sequential)**	1.067 ± 0.164	1.086 ± 0.141	1.245 ± 0.144	0.972 ± 0.137**%**	0.829 ± 0.102	0.868 ± 0.073	0.879 ± 0.124	0.814 ± 0.085
**Low BNZ + High Vaccine (sequential)**	1.132 ± 0.130	1.096 ± 0.173	1.216 ± 0.187	1.097 ± 0.228	0.858 ± 0.065	0.853 ± 0.137	0.865 ± 0.111	0.846 ± 0.102
**Curative BNZ**	1.164 ± 0.154	1.054 ± 0.081	0.944 ± 0.145*	0.873 ± 0.118**$	0.7901 ± 0.060	0.806 ± 0.074	0.763 ± 0.095	0.752 ± 0.064**$**

Left ventricular posterior wall thickness. The mean ± SD of left ventricular wall thickness was measured from M-mode echocardiography images during systole (LVPWs) and diastole (LVPWd). When comparing to 72 DPI, *≤0.05, **≤0.01, ****≤0.0001. When comparing to 122 DPI, $≤0.05,When comparing to 154 DPI %≤0.05.

**Table 2:** Left ventricular end diameter during systole and diastole.

	LVEDs				LVEDd			
Days post infection	72	122	154	208	72	122	154	208
**Naive**	3.081 ± 0.023	3.024 ± 0.277	3.000 ± 0.400	3.048 ± 0.0361	3.941 ± 0.195	3.969± 0.194	4.020± 0.307	4.013 ± 0.246
**Infected untreated**	2.288 ± 0.196	2.411 ± 0.527	2.542 ± 0.305	2.795 ± 0.401	3.575 ± 0.100	3.672 ± 0.395	3.724 ± 0.188	3.886 ± 0.347
**Low BNZ**	2.146 ± 0.573	2.643 ± 0.424	2.570 ± 0.370	2.582 ± 0.294	3.425 ± 0.639	3.893 ± 0.217	3.859 ± 0.322	3.825 ± 0.291
**Low E6020**	2.569 ± 0.038	2.290 ± 0.554	2.630 ± 0.326	3.081 ± 0.619	3.829± 0.334	3.570± 0.384	3.976± 0.300 **$$**	4.263± 0.599 **$$**
**Low Vaccine (sequential)**	2.352 ± 0.543	2.624 ± 0.479	2.443 ± 0.519	2.608 ± 0.226	3.789 ± 0.295	3.783 ± 0.305	3.581 ± 0.311	3.723 ± 0.239
**Low BNZ + Low Vaccine (sequential)**	2.374 ± 0.401	2.652 ± 0.621	2.753 ± 0.286 *	2.895 ± 0.351 *	3.573 ± 0.266	3.719 ± 0.317	3.915 ± 0.180 **	3.959 ± 0.271 *
**Low vaccine (concurrent)**	2.379 ± 0.485	2.481 ± 0.487	2.694 ± 0.425	2.576 ± 0.446	3.676 ± 0.323	3.712 ± 0.381	3.995 ± 0.335 *	3.819 ± 0.310
**Low BNZ + Low Vaccine (concurrent)**	2.190 ± 0.539	2.497 ± 0.475	2.550 ± 0.163	2.724 ± 0.393 *	3.618 ± 0.297	3.641 ± 0.183	3.761 ± 0.222	3.911 ± 0.215 **$**
**High E6020**	2.272 ± 0.395	2.634 ± 0.577	2.366 ± 0.385	2.613 ± 0.541	3.507 ± 0.257	3.784 ± 0.364	3.729 ± 0.248	3.723 ± 0.350
**High Vaccine (sequential)**	2.596 ± 0.343	2.633 ± 0.509	2.409 ± 0.378	2.783 ± 0.363	3.725 ± 0.267	3.7740± 0.370	3.770 ± 0.344	3.846 ± 0.220
**Low BNZ + High Vaccine (sequential)**	2.581 ± 0.279	2.563 ± 0.388	2.363 ± 0.341	2.565 ± 0.378	3.768 ± 0.197	3.713 ± 0.259	3.730 ± 0.228	3.797 ± 0.192
**Curative BNZ**	2.109 ± 0.398	2.621 ± 0.384 **	2.932 ± 0.400 **	3.039 ± 0.341 **	3.476 ± 0.269	3.695 ± 0.266***	4.007 ± 0.271**	4.093 ± 0.262**$

Left ventricular end diameter during systole and diastole. The mean ± SD of LVED was measured from M-mode echocardiography images at the end of systole (LVEDs ) and diastole (LVEDd). When comparing to 72 DPI, *≤0.05, **≤0.01. ***≤0.001. When comparing to 122 DPI, $≤0.05, $$≤0.01.

**Table 3:** Left ventricular volume.

	ESV				EDV			
Days post infection	72	122	154	208	72	122	154	208
**Naive**	37.66 ± 6.66	36.15 ± 7.88	35.95 ± 11.02	37.13 ± 10.35	67.79 ± 8.00	68.95 ± 8.13	71.41 ± 12.75	70.91 ± 10.64
**Infected untreated**	18.12 ± 4.06	21.90 ± 11.79	23.77 ± 7.11	30.32 ± 10.72	53.60 ± 3.61	58.00 ± 14.29	59.23 ± 7.04	66.08 ± 14.14
**Low BNZ**	17.00 ± 9.79	26.68 ± 10.10 *	24.73 ± 8.73	24.70 ± 7.18	50.78 ± 18.45	66.59 ± 9.15	67.80 ± 11.03	65.13 ± 12.54
**Low E6020**	24.62 ± 8.62	19.61 ± 12.11	25.93 ± 7.63	39.56 ± 20.41	63.77 ± 12.98	55.14 ± 14.24	69.54 ± 12.48	83.57 ± 30.67
**Low Vaccine (sequential)**	20.74 ± 10.09	26.19 ± 11.92	22.52 ± 10.93	25.12 ± 5.14	62.05 ± 11.25	61.85 ± 11.80	54.31 ± 11.15	59.37 ± 8.80
**Low BNZ + Low Vaccine (sequential)**	20.55 ± 8.487	28.00 ± 14.07	28.84 ± 7.11	32.81 ± 9.55 *	53.45 ± 10.11	59.29 ± 11.96	66.35 ± 7.41 *	69.25 ± 11.67 *
**Low vaccine (concurrent)**	21.11 ± 10.74	23.31 ± 11.13	27.94 ± 9.90	25.22 ± 10.35	57.9 ± 12.39	59.46± 14.99	70.52± 13.78 *	63.33 ± 12.11
**Low BNZ + Low Vaccine (concurrent)**	18.11 ± 8.39	23.38 ± 10.81	22.32 ± 5.83	29.14 ± 8.69 *	55.63± 10.71	56.14± 6.76	60.74± 7.83	66.68 ± 8.85 **$**
**High E6020**	18.41 ± 7.61	27.25 ± 14.18	20.24 ± 8.53	26.44 ± 12.28	51.51± 9.08	62.12 ± 13.78	59.58± 9.53	59.68 ± 13.20
**High Vaccine (sequential)**	25.21 ± 7.87	26.4 ± 11.45	21.15 ± 8.05	29.87 ± 9.48	59.52 ± 10.15	61.83 ± 14.11	61.49 ± 13.45	64.05 ± 8.84
**Low BNZ + High Vaccine (sequential)**	24.64 ± 6.35	23.58 ± 8.50	20.03 ± 7.13	24.63 ± 8.91	60.97 ± 7.54	57.97 ± 9.63	59.61 ± 8.62	62.07 ± 7.56
**Curative BNZ**	15.44 ± 7.10	25.97 ± 9.49 **	34.38 ± 11.68 **	38.32 ± 11.98 **	50.48 ± 9.56	58.36 ± 9.56 **	71.04 ± 11.39 **	74.38 ± 11.20 ** $

Left ventricular volume. The mean± SD of left ventricular volume was measured from M-mode echocardiography images at the end of systole (ESV ) and diastole (EDV). When comparing to 72 DPI, *≤0.05, **≤0.01. When comparing to 122 DPI, $≤0.05.

**Figure 6:**
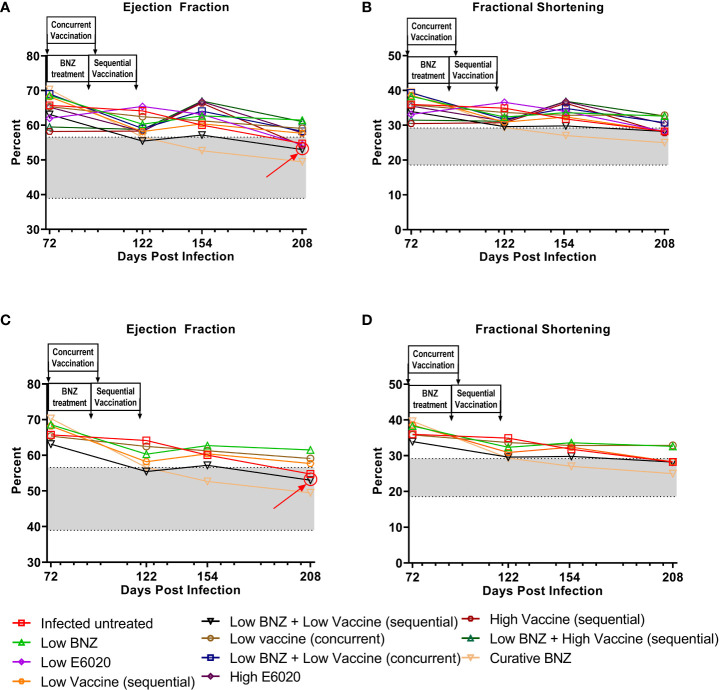
Ejection fraction and fractional shortening after treatment. M-mode images from echocardiographic examination were used to measure left ventricular ejection fraction (EF, panel **A**) and fractional shortening (FS, panel **B**) in naïve, infected untreated and treated mice at 70dpi, 122dpi, 154dpi and 208dpi. Panels **(C)** and **(D)** illustrate only a subset of treatment groups to better visualize treatment differences between key groups. The grey shaded area represents the mean ± SD of the EF and FS measurements from naïve age matched controls.

We also evaluated the impact of treatment on the *T. cruzi* induced changes in cardiac function by measuring EF and FS. Both sequential and concurrent treatment with Low BNZ + Low Vaccine, as well as the high dose vaccine alone significantly reduced EF at 208 DPI (**Figure 6**, **Table 4**). Curative BNZ treatment significantly reduced EF at 122 DPI, 154 DPI and 208 DPI (**Figure 6**, **Table 4**). Similarly, curative BNZ treatment significantly reduced FS at 122 DPI, 154 DPI and 208 DPI (**Figure 6**, **Table 4**). Cardiac function was also evaluated using electrocardiograms, but very few arrhythmias were observed regardless of treatment group (data not shown), thus we were unable to determine if treatments resulted in statistically significant differences in the incidence of arrhythmias. Together these data suggest that vaccine-linked chemotherapy can improve infection induced changes in cardiac function.

**Table 4:** Left ventricular ejection fraction and fractional shortening.

	EF				FS			
Days post infection	72	122	154	208	72	122	154	208
**Naive**	44.54 ± 6.77	47.57 ± 10.25	50.59 ± 9.12	48.33 ± 9.02	21.83 ± 3.94	23.81 ± 6.10	25.62 ± 5.57	24.24 ± 5.55
**Infected untreated**	65.73 ± 8.18	64.14 ± 12.44	60.03 ± 9.89	54.76 ± 9.35	35.96 ± 5.59	34.9 ± 8.39	31.78 ± 6.90	28.23 ± 6.10
**Low BNZ**	68.59 ± 11.26	60.30 ± 12.29	62.70 ± 7.692	61.50 ± 5.54	38.28 ± 8.66	32.35 ± 8.52	33.62 ± 5.37	32.6 ± 4.05
**Low E6020**	62.01 ± 8.455	65.40 ± 17.56	63.10 ± 9.74	54.40 ± 9.10	33.12 ± 5.80	36.58 ± 12.21	33.95 ± 5.09	28.19 ± 5.97
**Low Vaccine (sequential)**	68.18 ± 12.26	58.19 ± 15.04	60.41 ± 13.53	57.69 ± 6.56	38.55 ± 10.66	30.88 ± 9.27	32.36 ± 9.63	28.27 ± 4.66
**Low BNZ + Low Vaccine (sequential)**	63.11 ± 9.94	55.44 ± 16.83	57.17 ± 7.79	53.00 ± 7.96*	33.93 ± 7.07	29.59 ± 12.16	29.79 ± 5.33	28.22 ± 7.87
**Low vaccine (concurrent)**	65.35 ± 10.94	62.48 ± 10.36	61.25 ± 9.42	59.08 ± 13.01	35.80 ± 8.06	33.65 ± 7.53	32.84 ± 6.91	32.86 ± 7.99
**Low BNZ + Low Vaccine (concurrent)**	69.01 ± 11.35	59.07 ± 16.32	64.05 ± 7.96	58.28 ± 9.50*	39.27 ± 11.30	31.72 ± 10.68	34.83 ± 5.54	30.72 ± 7.12
**High E6020**	65.20 ± 10.25	58.22 ± 14.99	66.80 ± 9.65	57.78 ± 12.43	35.49 ± 7.88	31.18 ± 9.44	36.77 ± 7.23	30.44 ± 8.70
**High Vaccine (sequential)**	58.22 ± 9.13	58.31 ± 11.33	66.37 ± 7.89	54.14 ± 9.77*	30.46 ± 6.19	30.74 ± 7.72	36.32 ± 5.87	27.83 ± 6.17**%**
**Low BNZ + High Vaccine (sequential)**	59.52 ± 9.68	58.77 ± 12.37	66.97 ± 8.13	61.04 ± 10.83	31.47 ± 6.71	31.11 ± 8.23	36.86 ± 6.36**$**	32.65 ± 7.53
**Curative BNZ**	70.31 ± 10.25	56.58 ± 9.26**	52.65 ± 10.80*	49.5 ± 9.21**	39.62 ± 8.39	29.33 ± 6.10**	27.03 ± 6.74*	24.98 ± 5.50**

The mean± SD of left ventricular ejection fraction (EF) and fractional shortening (FS) was measured from M-mode echocardiography. When comparing to 72 DPI, *≤0.05, **≤0.01. ***≤0.001, ****≤0.0001. When comparing to 122 DPI, **$**≤0.05,When comparing to 154 DPI **%**≤0.05

### Vaccine-linked chemotherapy induces a balanced antigen specific T_H_1/T_H_2 response

**Figure 7:**
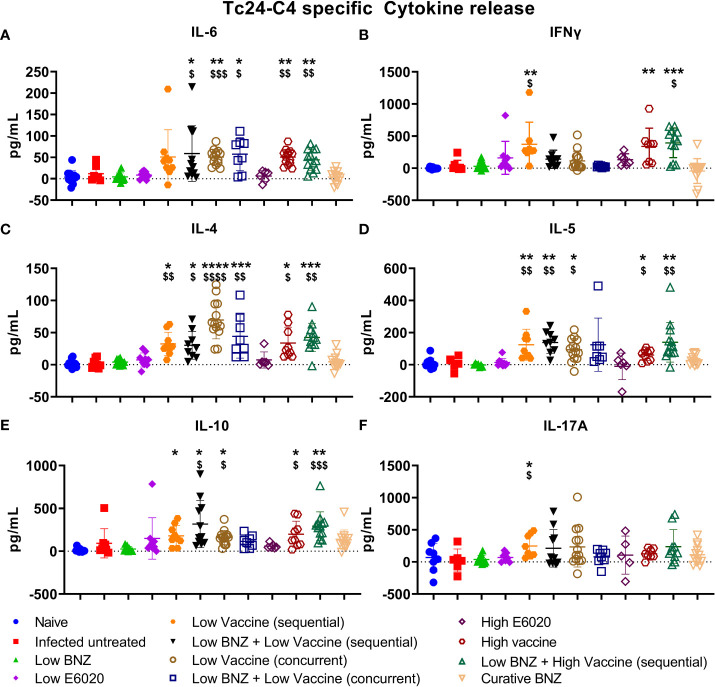
Antigen specific TH1/TH2/TH17 cytokine release from splenocytes. Splenocytes were restimulated in vitro with recombinant Tc24-C4 protein or cRPMI alone (unstimulated) as described. Culture supernatants were harvested after 96 hours and stored at -80C until quantification of cytokines by Luminex assay. Data was analyzed for normality, then treatment groups were compared to controls using parametric or non-parametric ANOVA depending on normality. When comparing groups to the infected untreated control *, p≤0.05; **, p≤0.01; ***, p≤0.001; ****, p≤0.0001. When comparing groups to E6020 only treatment $, p≤0.05. $$, p≤0.01; $$$, p≤0.001; $$$$, p≤0.0001.

We previously demonstrated that vaccine-linked chemotherapy during acute *T. cruzi* infection, combining low dose BNZ with either low or high dose vaccines containing the Tc24-C4 protein antigen and a TLR-4 agonist adjuvant induced a balanced T_H_1/T_H_2/T_H_17 cytokine response with significant increases in antigen specific IFN-γ, IL-4, and IL-17A release from splenocytes (Cruz-Chan et al., 2021). Here we evaluated cytokine release from splenocytes harvested from mice treated during chronic *T. cruzi* infection. Treatment with Low BNZ + Low Vaccine resulted in significantly increased IL-6 (**Figure 7A**), IL-4 (**Figure 7C**), IL-5 (**Figure 7D**), and IL-10 (**Figure 7E**). Similarly, treatment with Low BNZ + High Vaccine resulted in significantly increased IL-6 (**Figure 7A**), IFNγ (**Figure 7B**), IL-4 (**Figure 7C**), IL-5 (**Figure 7D**), and IL-10 (**Figure 7E**). When evaluating vaccines alone, Low Vaccine (sequential) induced significantly increased IFNγ (**Figure 7B**), IL-4 (**Figure 7C**), IL-5 (**Figure 7D**), IL-10 (**Figure 7E**) and IL-17A (**Figure 7F**), while both Low Vaccine (concurrent) and High Vaccine induced significantly increased IL-6 (**Figure 7A**), IL-4 (**Figure 7C**), IL-5 (**Figure 7D**), and IL-10 (**Figure 7E**). Together these data indicate that the Tc24-C4/E6020 SE vaccine at is driving a balanced T_H_1/T_H_2 antigen specific cytokine response, with modest IL-17A production, which does not appear to be affected by BNZ treatment.

**Figure 8:**
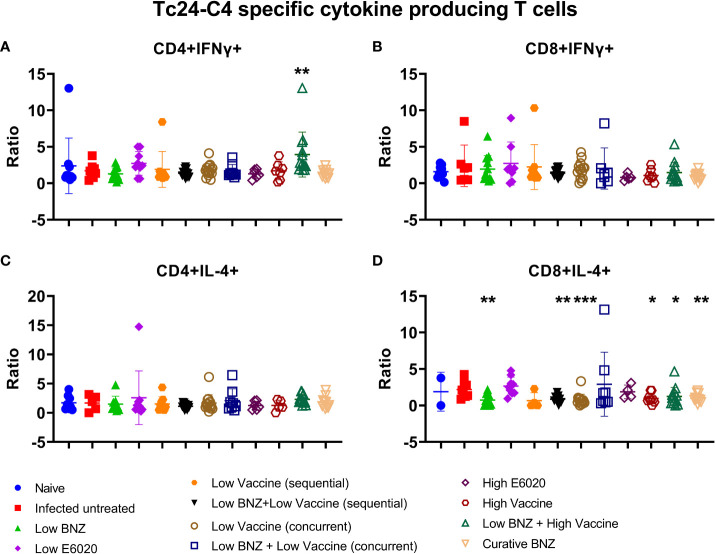
Antigen specific cytokine producing T cells. Splenocytes were restimulated in vitro with recombinant Tc24-C4 protein or cRPMI alone (unstimulated), then stained with fluorescent antibodies for analysis by flow cytometry as described. Results are expressed as a ratio of antigen specific cells divided by unstimulated cells. **(A)** CD4+IFNγ+ cells, **(B)** CD8+IFNγ+ cells, **(C)** CD4+IL-4+ cells, **(D)** CD8+IL-4+ cells. When comparing groups to the infected untreated control *, p≤0.05; **, p≤0.01; ***, p≤0.001.

To determine whether vaccines induced antigen specific cytokine production from T cells, IFNγ and IL-4 producing CD4+ and CD8+ T cells were analyzed by flow cytometry. Low BNZ + High vaccine induced significantly increased CD4+IFNγ+ T cells compared to infected untreated mice (**Figure 8A**). No treatments significantly affected CD8+IFNg+ T cells (**Figure 8B**). In contrast, multiple treatments caused significant reductions in the CD8+ IL-4+ population, including Low BNZ, Low BNZ + Low Vaccine (sequential), Low Vaccine (concurrent), High Vaccine, Low BNZ + High Vaccine, and Curative BNZ (**Figure 8D**). No treatments significantly affected CD4+IL-4+ cells (**Figure 8C**). This suggests that in mice treated with Low BNZ + High Vaccine, CD4+ T cells may be a key source of the increased IFNγ released from splenocytes restimulated *in vitro.*

### Therapeutic vaccination significantly reduced cardiac pathology

**Figure 9:**
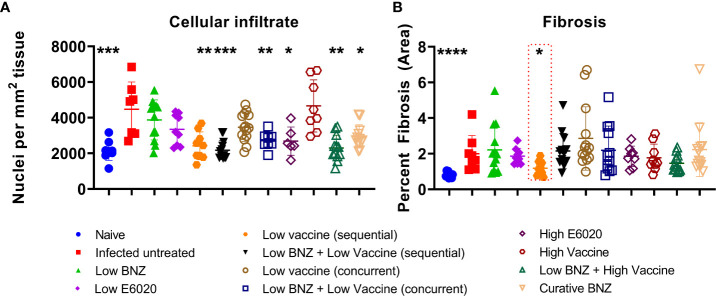
Cardiac Cellular infiltrate and fibrosis. Cardiac cellular infiltrate **(A)** was quantified from representative images of H&E stained tissue sections as described. Fibrosis **(B)** was quantified from representative images of Masson’s Trichrome stained tissue sections as described. When comparing groups to the infected untreated control *, p≤0.05; **, p≤0.01; ***, p≤0.001; ****, p≤0.0001.

**Figure 10:**
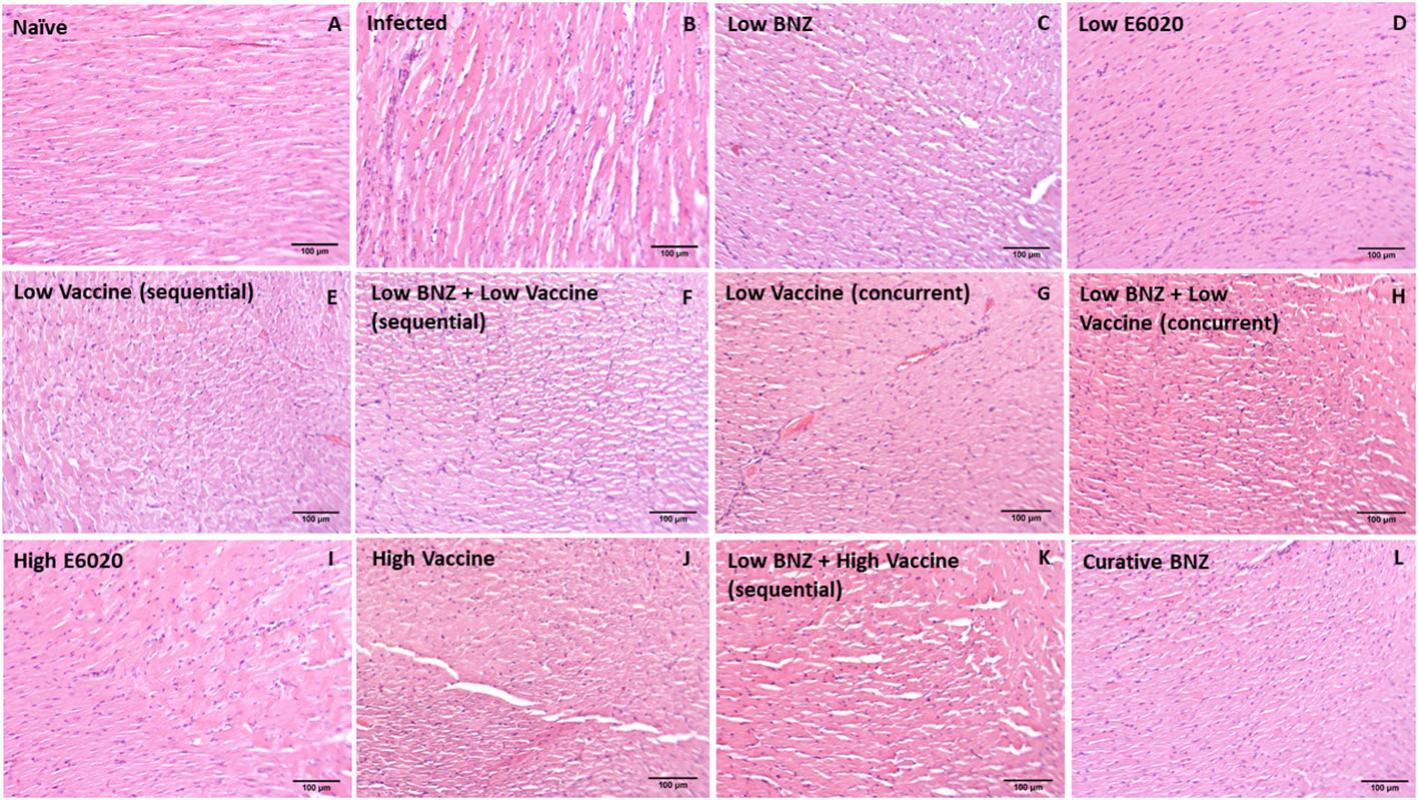
Representative images of H&E stained cardiac tissue sections from naïve **(A)**, infected untreated **(B)** and treated **(C–L)** mice. Images were captured at 100X magnification. Scale bar represents 100µm.

**Figure 11:**
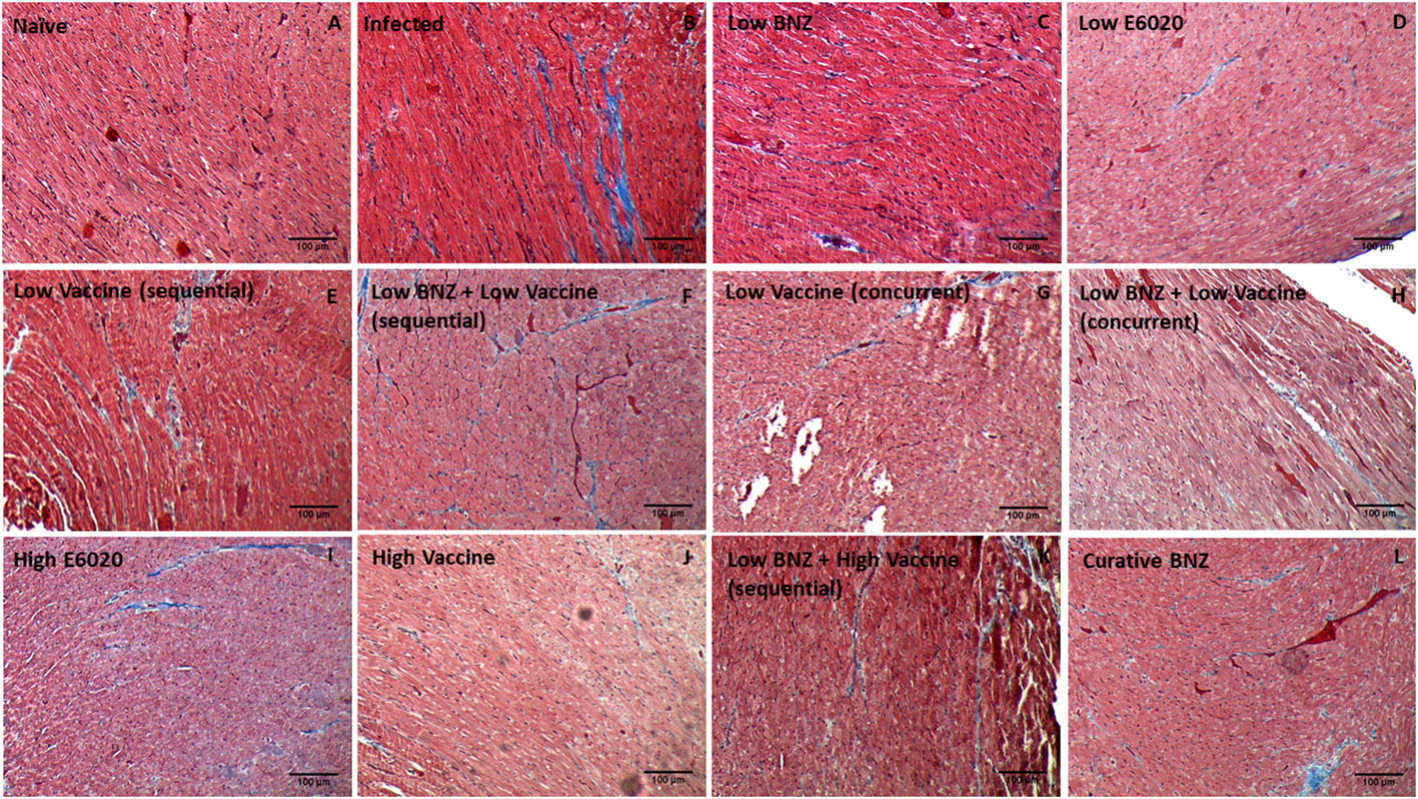
Representative images of Masson’s Trichrome stained cardiac tissue sections from naïve **(A)**, infected untreated **(B)** and treated **(C–L)** mice. Images were captured at 100X magnification. Scale bar represents 100µm.

Vaccine-linked chemotherapy using a low dose of the Tc24-C4/E6020 vaccine has been shown to significantly reduce *T. cruzi* induced cardiac fibrosis and inflammation in an acute model of infection (Jones et al., 2018; Cruz-Chan et al., 2021), and a higher dose of the Tc24-C4/E6020 vaccine alone significantly reduced fibrosis in a chronic model of infection (Barry et al., 2019). Recently, it was demonstrated that vaccine-linked chemotherapy using a bivalent vaccine containing both the Tc24-C4 and TSA1-C4 recombinant protein antigens combined with E6020 SE adjuvant significantly reduced cardiac fibrosis in a chronic model of infection (Dzul-Huchim et al., 2022). Here we evaluated the effect of our monovalent Tc24-C4/E6020 SE vaccine, either alone or in combination with Low BNZ, on cardiac fibrosis and inflammation. Both sequential and concurrent treatment with Low BNZ + Low Vaccine and Low BNZ + High Vaccine significantly reduced infiltration of cells into cardiac tissue, similar to the curative dose of BNZ (**Figures 9**, **10**). Additionally Low vaccine (sequential) alone, as well as the High E6020 alone significantly reduced cellular infiltrate. Low vaccine (sequential) significantly reduced the percent fibrosis in cardiac tissue when compared to infected untreated mice (**Figures 9B**, **11**). Together, this suggests that the monovalent Tc24-C4/E6020 SE vaccine, either alone or in combination with Low BNZ, significantly reduce cardiac inflammation and in the case of the Low vaccine (sequential) also reduce cardiac fibrosis. Cardiac parasite burdens were detectable, but below the limit of quantitation based on the standard curve used in this assay, thus differences based on treatment could not be determined (**Supplemental Figure 2**)

## Discussion

Chronic *T. cruzi* infection induces persistent inflammation, hypertrophy, and progressive fibrosis which leads to significant changes in cardiac structure and function, ultimately manifesting clinically as CCC (Rossi et al., 2003; Alvarez et al., 2014). Thus far, antiparasitic treatments alone are only able to eliminate, or significantly reduce, parasite burdens peripherally and do not prevent progression of clinical cardiac disease due to parasites in the cardiac tissue and resultant inflammation (Morillo et al., 2015). Here we have shown that our vaccine-linked chemotherapy (VLC) treatment regimens, combining a low dose BNZ treatment with our Tc24-C4 protein/TLR-4 agonist (E6020) SE adjuvant vaccine, significantly improves cardiac structure and function in a mouse model of chronic *T. cruzi* infection. This strategy also reduced cardiac pathology, as evidenced by significantly reduced cellular infiltration and reduced fibrosis. The vaccine induced a durable T_H_1/T_H_2 cytokine response at low doses, with modest IL-17A production, when administered alone or with BNZ treatment in both sequential (**Supplemental Figure 3A)** and concurrent (**Supplemental Figure 3B**) treatment schemes, as well as the high dose vaccine given after low BNZ treatment (**Supplemental Figure 3C**). These data suggest that modulation of the host immune response effectively ameliorates the deleterious host responses to *T. cruzi* infection that contribute to tissue pathology and clinical disease. The fact that these effects were observed with a low dose of BNZ combined with a low dose of vaccine, indicates that vaccine-linked chemotherapy is an effective dose-sparing strategy. These data support our prior findings in mouse models of acute infection which demonstrated that vaccine-linked chemotherapy showed improved efficacy compared to monotherapy with BNZ or the Tc24-C4/E6020 SE vaccine alone (Jones et al., 2018; Cruz-Chan et al., 2021). Importantly, our results agree with recently published results demonstrating efficacy of vaccine-linked chemotherapy in mouse models of chronic *T. cruzi* infection. Prochetto et al. showed that vaccination with three doses of a recombinant Trans-sialidase protein combined with ISPA adjuvant, followed by a curative course of BNZ led to reduced cardiac arrhythmias and fibrosis in chronically infected mice (Prochetto et al., 2022). Dzul-Huchim et al. observed significantly reduced cardiac fibrosis in chronically infected mice that were vaccinated with two doses of a vaccine containing both recombinant TSA-1 protein and Tc24-C4 protein combined with E6020 SE adjuvant concurrently with a 7 day course of low dose BNZ treatment (Dzul-Huchim et al., 2022). Altogether, these data provide proof-of-concept that combining BNZ with vaccines can effectively targeting both the parasite and the deleterious host immune responses and improve cardiac function. Additionally, in both the Dzul-Huchim et al. study and our study, we showed that combining multiple treatments can allow dose sparing of both BNZ and the vaccine, which will likely result in reduced incidence of adverse effects and improve tolerability of treatment.

The clinical manifestations of CCC are due to *T. cruzi* induced cardiac remodeling (Rossi et al., 2003; Alvarez et al., 2014; Noya-Rabelo et al., 2018; Senra et al., 2018). For example, dilated cardiomyopathy is a severe manifestation of end stage CCC characterized by significant thinning of ventricular walls, left ventricular enlargement, and significantly reduced ejection fraction (Nunes et al., 2013). We previously demonstrated in our mouse model that chronic infection leads to significant compromise of cardiac function as evidenced by significantly increased cardiac circumferential strain on MRI and echocardiography (Hoffman et al., 2019). However, in that study we did not objectively evaluate changes in cardiac structure with imaging modalities as infection progressed. In the study reported here, we evaluated left ventricular wall thickness, chamber size and volume over time in an effort to better characterize the changes in cardiac structure induced by chronic *T. cruzi* infection. Interestingly, we observed significantly increased LVPW thickness as early as 28 DPI, representing the acute stage of infection. While this wall thickening did naturally regress over time, by approximately 70 DPI it had not yet reached the thickness measured in age matched controls (**Figure 2A**). Further, LVPW thickness in infected untreated mice did not regress to levels similar to naïve age matched controls until approximately 208 DPI (**Figures 3A, B**). The increased LVPW thickness induced by *T. cruzi* infection was accompanied by reductions in left ventricular diameter (**Figure 4**) and volume (**Figure 5**) at the end of both systole and diastole, but an increase in ejection fraction (**Figure 6A**). This suggests that *T. cruzi* infection can lead to reduced filling of the left ventricle, which may compromise effective tissue perfusion thus the increase in ejection fraction may be a compensatory mechanism to preserve cardiac output. In our previous study characterizing cardiac health and fibrosis during chronic infection, we observed that while all infected mice had increased cardiac strain, only a portion of mice concurrently had reduced ejection fraction while the remaining mice had preserved ejection fraction (Hoffman et al., 2019). This suggests that in our model the mice are developing heart failure with preserved ejection fraction (HFpEF). In HFpEF, it is proposed that systemic inflammation causes microvascular inflammation leading to cardiomyocyte hypertrophy and interstitial fibrosis, resulting in left ventricular stiffness and heart failure (Paulus and Tschöpe, 2013). Further, in HFpEF thickening of the left ventricular wall is accompanied by increased wall stiffness and more forceful contraction, resulting in higher than normal EF in order to preserve cardiac output (Dori et al., 2012). CCC is an inflammatory cardiomyopathy due to parasite persistence, and has a worse prognosis than non-inflammatory cardiomyopathies (Higuchi et al., 1987; Bestetti and Muccillo, 1997). Considering this, we propose that in our model *T. cruzi* induced inflammation resulted in HFpEF with increased EF and LVPW thickness measured on echocardiography,and increased cardiac fibrosis on histopathology. Additionally, a balanced T_H_1/T_H_2/T_H_17 immune response is associated with better cardiac function in individuals in the indeterminate stage of Chagas disease (Sousa et al., 2014). Here we report that the Tc24-C4/E6020 SE vaccine induced increased antigen specific release of IL-6, IFNγ, IL-4, IL-5, IL-10, and IL-17A. Taking these findings into account, we propose that efficacy of our VLC strategy is due in part to induction of a balanced antigen specific T_H_1/T_H_2/T_H_17 immune response by the vaccine which results in reduced cardiac inflammation and fibrosis, leading to reduced left ventricular posterior wall thickness and reduced EF, and ultimately preservation of cardiac function.

Cardiac fibrosis is a consistent finding in CCC and a major driver of clinical disease (Marin-Neto et al., 2007; Senra et al., 2018). Even in indeterminate patients, cardiac fibrosis is detectable with advanced imaging techniques (Macedo et al., 2015). In preclinical models of acute and chronic *T. cruzi* infection, therapies that interrupt key promoters of fibrosis, including TGFβ and pY-STAT3, do effectively reduce cardiac fibrosis (Waghabi et al., 2009; de Oliveira et al., 2012; Hoffman et al., 2021). Additionally, we have previously shown that Tc24 based vaccines when used alone or in a VLC strategy significantly reduce cardiac fibrosis in both acute and chronic mouse models of infection (Barry et al., 2019; Cruz-Chan et al., 2021; Dzul-Huchim et al., 2022). We reported here that while the low dose vaccine alone was able to significantly reduce endpoint fibrosis compared to infected untreated controls, statistically significant reductions in fibrosis were not observed in any groups that received VLC (**Figure 9B** and **Figure 11**). This may be due to differences in the vaccination doses, timing of vaccination, or our use of a monovalent vaccine compared to the bivalent vaccine used by Dzul-Hchim et al. This also suggests that while VLC is partially efficacious, additional targeting of the host fibrotic response to *T. cruzi* is necessary to improve treatment efficacy. We have previously shown that by inhibiting the activity of pY-STAT3 with a targeted inhibitor, TTI-101, we significantly reduced cardiac fibrosis and serum levels of TGFβ and PDGF-D without affecting tissue parasite burdens (Hoffman et al., 2021). However, since pY-STAT3 regulates inflammatory responses, both by blocking IFNγ induced STAT1 and IL-6 induced NFκB (Hovsepian et al., 2013; Yu et al., 2017), inhibiting pYSTAT3 exacerbated cardiac inflammation (Hoffman et al., 2021). In the Hoffman et al. study, as well as in the study reported here, we observed that while treatment with a curative dose of BNZ alone did not significantly reduce cardiac fibrosis, it did reduce cardiac inflammation. Thus, we propose that we could improve treatment efficacy by first using a treatment to significantly reduce cardiac inflammation, then follow with a targeted anti-fibrotic treatment, such as TTI-101. Studies are ongoing to evaluate whether such a sequential, multi-modal treatment strategy would better resolve *T. cruzi* induced cardiac pathology.

Limitations of the study reported here have been identified and are under careful consideration to better inform the design of future studies. Here we demonstrated that chronic *T. cruzi* H1 infection induced significant changes in cardiac structure and function in female BALB/c mice that were ameliorated with VLC. We elected to use female mice only because we have previously demonstrated that *T. cruzi* H1 infection induces characteristic cardiac pathology and changes in cardiac function in both acutely and chronically infected female BALB/c mice (Jones et al., 2018; Hoffman et al., 2019; Cruz-Chan et al., 2021; Hoffman et al., 2021). Additionally, we demonstrated initial proof-of-concept for therapeutic efficacy of our VLC strategy using acutely infected female mice (Jones et al., 2018; Cruz-Chan et al., 2021). Therefore we elected to use our established female BALB/c mouse model of chronic *T. cruzi* H1 strain infection to evaluate efficacy of our VLC strategy initiated during chronic infection. However, some studies have shown that male Chagas patients have a higher incidence of developing cardiac disease compared to females (Sabino et al., 2013). Therefore we are planning future studies with both male and female mice to evaluate VLC efficacy in chronically infected male mice, and to determine if efficacy is comparable between the sexes. An additional limitation is that we only evaluated left ventricular wall thickness, diameter, volume, ejection fraction and fractional shortening to determine the effects of VLC in our chronic infection mouse model. Evaluation of circumferential strain, as performed in prior studies, would have allowed us to determine if increased left ventricular wall thickness correlated with increased circumferential strain, further supporting our hypothesis that cardiac disease in our model can be characterized as HFpEF. We only observed significant reductions in fibrosis in the Low Vaccine (sequential) group and a trend toward reduced fibrosis in the Low BNZ + High Vaccine group (p=0.1403). In a prior study where we observed significantly reduced fibrosis in chronically infected mice treated with Tc24/E6020 SE vaccine mice were evaluated at approximately 180 DPI (Barry et al., 2019), compared to approximately 208 DPI as in the study reported here. Thus, in future studies we will evaluate the effect of treatment on cardiac fibrosis at earlier timepoints to determine if treatment efficacy may wane with the current strategy, and additional vaccine doses may be needed to improve durability of the effect. In this study, while we showed that the Tc24-C4/E6020 SE vaccine induced the desired T_H_1/T_H_2/T_H_17 cytokine profile that has been associated with less clinical disease in indeterminate individuals (Sousa et al., 2014), we did not show any significant increases in antigen specific CD8+ cytokine producing T cells with the vaccine. This is consistent with what was observed by Dzul-Huchim et al, who also showed that VLC induced increased CD4+ and CD8+ perforin producing effector T cells (Dzul-Huchim et al., 2022). In the study reported here we did not evaluate functional activity of effector cells, including production of perforin and granzyme, thus it will be important to evaluate effector cell functions in addition to cytokine producing cells in future studies. This may also correlate with the low parasite burden detected. Further, it will be important in future studies to immunophenotype cardiac derived immune cells, and measure cardiac specific cytokine production and expression of inflammatory markers, such as STAT1 and NFκB. This knowledge would better define disease pathogenesis and mechanisms of protection induced by the VLC strategy.

The etiology of Chagas disease was fully described over 100 years ago, but there are as yet only two approved treatments for the disease with limited efficacy during chronic infection and high rates of adverse side effects (Lewinsohn, 1979; Viotti et al., 2009; Jackson et al., 2010). Several studies have focused on identifying new antiparasitic drugs, testing new drug combinations, and developing new dosing strategies in an effort to improve efficacy and tolerability (Perez-Mazliah et al., 2013; Bustamante et al., 2014; García-Huertas and Cardona-Castro, 2021). Additionally, dozens of candidate vaccines have been designed and tested in pre-clinical models, but none have yet reached testing in human clinical trials (Rios et al., 2019; Bivona et al., 2020). Due to the complex nature of disease pathogenesis, therapies that target only the parasite or only the host response are unlikely to have high efficacy. Vaccine-linked chemotherapy has been proposed as an effective strategy to reduce both infection and disease burdens for parasitic diseases, including schistosomiasis and malaria (Bergquist et al., 2005; von Seidlein et al., 2020). Thus, we have designed vaccine-linked chemotherapy strategy to both reduce parasite burdens and ameliorate the deleterious host responses that contribute to clinical cardiac disease. Here we provide important evidence that such a strategy will help bridge the current tolerability and efficacy gaps of the current single drug standard of treatment, and alleviate disease and disability of millions of Chagas patients worldwide.

## Data availability statement

The original contributions presented in the study are included in the article/**Supplementary Material**. Further inquiries can be directed to the corresponding author.

## Ethics statement

The animal study was reviewed and approved by Baylor College of Medicine’s Institutional Animal Care and Use Committee.

## Author contributions

KJ, MB, and PH designed the study. KJ, EM, LEV, JC, LV, BK, and AK acquired the data. JP and FG provided reagents. KJ, CR, LEV, and LV analyzed the data. KJ drafted the manuscript. All authors critically reviewed and revised the manuscript. All authors contributed to the article and approved the submitted version.
