# Supplementary material for: Vaccine-linked chemotherapy improves cardiac structure and function in a mouse model of chronic Chagas disease
Source: Front Cell Infect Microbiol. 2023 Feb 9;13:1106315. doi: 10.3389/fcimb.2023.1106315 (PMC9947347; doi:10.3389/fcimb.2023.1106315)
Supplement: Supplementary file 1 [file DataSheet_1.pdf]

Supplemental Data:

Title: Vaccine-linked chemotherapy improves cardiac structure and function in a mouse model of chronic Chagas disease.

Authors: Kathryn M. Jones[1,2*], Elise N. Mangin[3], Corey L. Reynolds[3], Liliana E. Villanueva[1,4], Julio Vladimir Cruz[1,4], Leroy Versteeg[1,5], Brian Keegan[1], April Kendricks[1], Jeroen Pollet[1], Fabian Gusovsky[6], Maria Elena Bottazzi[1,2,7], Peter J. Hotez[1,2,,7,8,9]

[1] Texas Children's Hospital Center for Vaccine Development, Department of Pediatrics, Division of Tropical Medicine, Baylor College of Medicine, Houston, Texas, United States of America

[2] Department of Molecular Virology and Microbiology, Baylor College of Medicine, Houston, Texas, United States of America

[3] Department of Molecular Physiology, Baylor College of Medicine, Houston, Texas, United States of America

[4] Laboratorio de Parasitología, Centro de Investigaciones Regionales Dr. Hideyo Noguchi, Universidad Autónoma de Yucatán, Mérida, Yucatán, México.

[5] Cell Biology and Immunology Group, Wageningen University & Research, De Elst 1, 6708 WD Wageningen, The Netherlands

[6] Global Health Research, Eisai, Inc., Cambridge, MA, United States of America

[7] Department of Biology, Baylor University, Waco, Texas, United States of America.

[8] James A. Baker III Institute for Public Policy, Rice University, Houston, Texas, United States of America

[9] Hagler Institute for Advanced Study at Texas A&M University, College Station, Texas, United States of America

*Corresponding author email kathrynj@bcm.edu

Keywords

Trypanosoma cruzi, Chagas disease, vaccine-linked chemotherapy, cardiomyopathy, cardiac function, fibrosis, inflammation

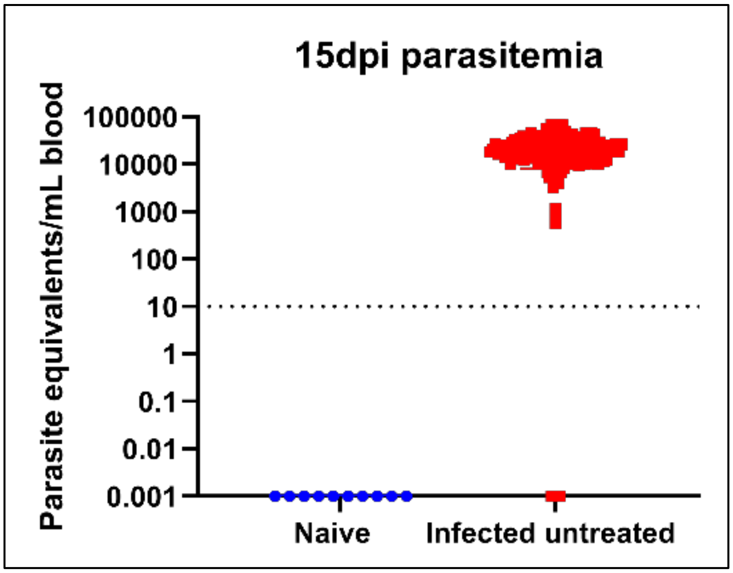

Supplemental Figure 1: Parasitemia to confirm infection. Parasitemia was measured from blood collected at 15dpi to confirm infection by qPCR as described. Any infected mice that were negative on qPCR were removed from the study.

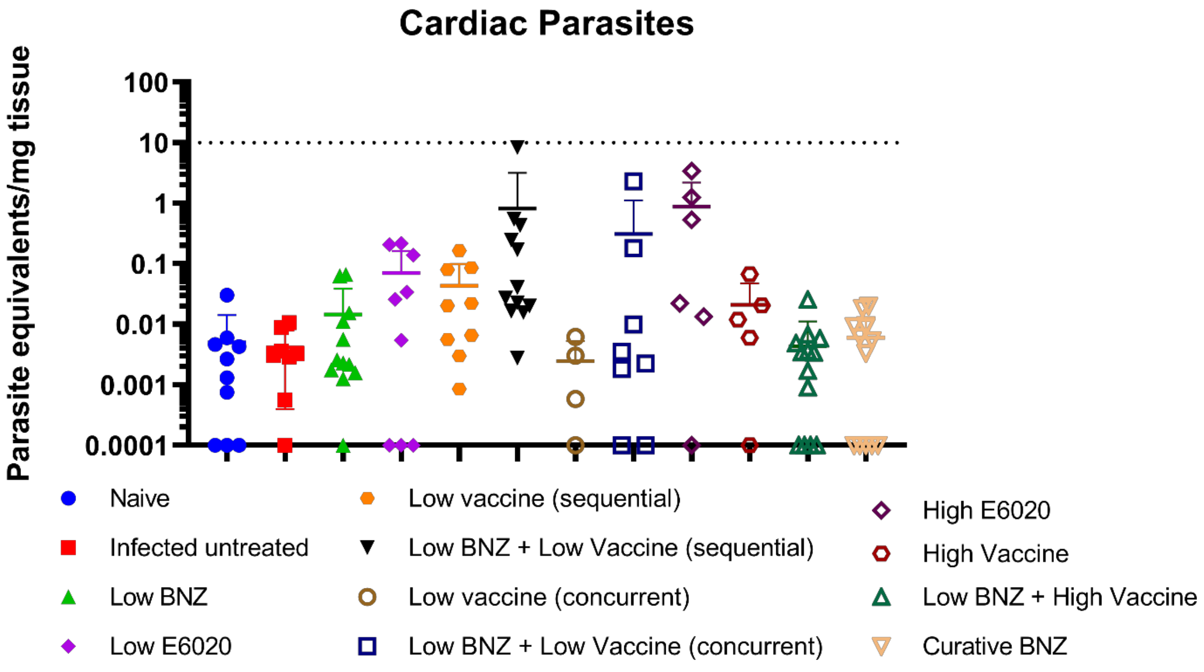

Supplemental Figure 2: Endpoint cardiac parasite burdens. Cardiac parasite burdens were measured from total DNA isolated from heart tissue sections as described. The dotted line represents the lower limit of quantitation from the standard curve used.

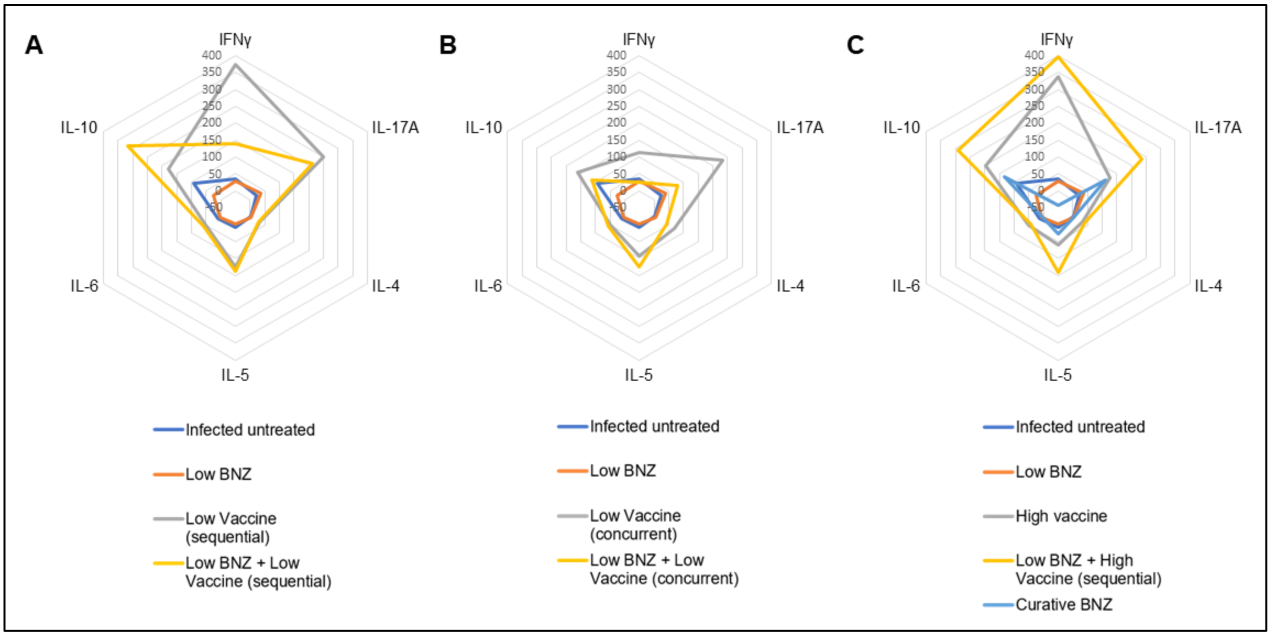

Supplemental Figure 3: Radar plots of antigen specific cytokine release. $T_H1/T_H2/T_H17$ cytokines were measured from supernatant of splenocytes restimulated with recombinant Tc24-C4 protein as described. The combined released cyokine pattern of groups treated with Low BNZ and Low Vaccine sequentially(Panel A), Low BNZ and Low Vaccine concurrently (Panel B) and Low BNZ and High Vaccine sequentially, with relevant controls, are plotted as radar graphs.
